# Premature Rupture Fetal Membranes

**Published:** 1873-12

**Authors:** Thomas Chesnut


*AMERICAN PRACTITIONER*, *December*, 1873.

Premature Rupture of the Fetal Membranes—*By Thomas Chesnut, M.D.*—I desire to present to the consideration of the profession the subject of rupture of the fetal membranes, and consequent escape of the *liquor amnii*, prior to the completion of pregnancy, and the treatment of cases in which this accident occurs.

In my first case, the patient, while walking, was surprised on finding an escape of water from her vagina—a quart as she supposed. The accident occurred toward the latter part of the seventh month of gestation. She had no pain, and no impression as yet having been made upon the os uteri, I gave her a quieting draught, left directions for the night, and returned home. On calling the next morning I found my patient calm and quite cheerful, having passed the night without pain or much uneasiness.

I then directed her to remain in bed, keep as quiet and composed as possible, and on the slightest approach of pain or uneasiness in the uterine region, to take a portion of a solution of morphine which I had prepared for that purpose, repeating the dose as occasion might require. This treatment of rest and morphine, occasionally alternated with lavements of laudanum and starch-water, was kept up; her bowels during the time being regulated by mucilaginous injections, until the twenty-third day after the accident, at which time expulsive pains ensued, and after a comparatively short and easy labor she was delivered of a living female child, bearing unmistakable marks of having made its appearance a little ahead of time. The child, though feeble, from the first nursed its mother, and after three or four weeks commenced to gain in flesh and grow rapidly. The patient's recovery was of the most favorable kind, this being her fourth confinement.

My second case was near the middle of the seventh month of her second pregnancy, at which time she was much surprised on finding a free escape of water from her vagina. I advised her to remain in bed and keep as quiet and composed as possible, and on the first approach of pain or uneasiness to take a full portion of an anodyne mixture which was ordered, and continue its use as occasion might require for subduing nervous restlessness and assuaging pain; at the end of forty-three days her labor commenced, and everything being favorable, she was soon delivered of a living male child.

The third, last, and most difficult one to control in her first pregnancy aborted between the third and fourth month. In her second, a twin case, premature labor occurred between the seventh and eigth month, and both children were still-born. In her third pregnancy, both parents being exceedingly anxious for a child, she was at the close of the sixth month placed under my care, with a special request to conduct her, if possible, safely to her full term. Supposing from her past history that she had the misfortune to possess a uterus more than ordinarily irritable, I recommended the strict observance of such precautionary measures as in my judgment were calculated to guard against a repetition of former misfortunes. Up to near the close of the seventh month her condition was quite favorable; then one day, while engaged in active work, and ascending a stairs carrying a heavy weight, she suddenly felt, as she said, "something give way and a splash of water on the floor." The result was, as might be expected, alarm attended with a paroxysm of nervous agitation. On my arrival gave her an opiate, assuring her at the time that there was still hope of saving the life of her child, if she would patiently submit to such means and restrictions as her condition might from time to time require. She readily consented, and for three consecutive weeks she was confined to her bed, and kept almost constantly under the influence of laudanum, as it acted most happily in tranquilizing her nervous system and obviating pain. The necessity for keeping her so constantly under the influence of the drug arose from the fact that as soon as she was permitted to pass from under its influence slight pains, with an increased flow would at once ensue. Up to this time she was not permitted to sit up in bed, and was required to use the bed-pan instead of the chamber. Having thus reached the latter part of the eighth month, she was permitted every morning to exchange for a brief time the bed for a lounge.

In precisely one week from the time this indulgence was granted, twenty days after rupture of membranes, I was summoned, and after a short and easy labor she was delivered of a living male child. This, though not more than an eight-month's child, was rather larger and more vigorous than either of the other two. It soon took the breast, from which it has received an abundant supply ever since. The patient suffered no particular inconvenience from the long-continued use of opium, and had a favorable recovery. The child, after the first month, began to improve, and at the present time is healthy and bright.

In none of the forgoing cases was there any protrusion of the membranes; nor did a careful digital exploration, especially in the last two cases, reveal either a perforation or rent in the membranes, and but little fluid escaped at the time of delivery. I am not ignorant of the fact that the possibility of prolonging gestation for any considerable length of time after a rupture of the fetal membranes has taken place is denied by authors of the very highest repute.

Ramsbotham remarks: "If, by any accident, the ovular membranes become ruptured, gastation is suspended and abortion necessarily ensues. At any period of pregnancy then a puncture through the chorion and amnion will sooner or later occasion the premature evacuation of the uterus." Again he remarks: "Cases indeed are reported in which it is supposed that a laceration of the involucra had taken place, and had subsequently become closed, the womb retaining its burden until the period was fulfilled. I am much inclined to the belief that in these instances some mistake had occurred, and error might easily arise from two, if not more, causes."

The causes here referred to, in the first place, is the occasional accumulation of a limpid serous fluid, contained between the two ovular membranes, resembling precisely in appearance the liquor amnii. In such case the chorion might rupture, giving vent to the fluid, while the amnion remained intact without seriously interfering with the process of gestation; and second, a morbid condition of the glandulæ nabothi, which occasionally occurs, producing a copious discharge of a serous secretion, which may escape in gushes, giving a deceptive idea of the true character of the discharge. But, as if determined that his views on this subject should be fully understood, he further remarks: "But although we are liable to these sources of error, I am persuaded that if both membranes be broken, and the least drop of water contained within the amnion flows away, the process of gestation is interrupted to such an extent as to insure the commencement of uterine contraction within a short time."

As neither of the causes assigned by that eminent author as a reason sufficient to account for the free escape of the fluid from the vagina without interrupting the process of gestation would properly apply to the cases above reported, as they would fail to afford a reasonable cause for the absence of the liquor amnii, which was found to be the condition in each case, this question can only be settled by repeated experiments and careful observation. And as the experiment would not to any considerable extent increase the dangers of the mother, and even if it did, surely no female possessed of the proper feelings of a mother would hesitate for a moment to take the risk, if by so doing she could greatly increase the chances of her child's life. It is not to be expected that every case, or even a large majority of the cases belonging to this class of accidents, can be protracted to a period of safety for the infant; but, judging from my own experience, I feel fully persuaded that a sufficient number of cases can be saved to insure the practice a respectable position among the more important contributions to conservative midwifery, if the profession can be induced to give the subject the attention that its importance demands.

*Editorial Remarks.*—Whatever any physician may believe as to the rupture of the fetal membranes weeks prior to the occurrence of labor, all will agree as to the practical value of the advice given by Dr. Chestnut in his interesting communication.

As will be seen in the concluding portion of the extract which we shall give from one of the most eminent of French obstetricians, Depaul, the purpose and part of the method of treatment of the two are the same, though our Indiana friend and the Paris professor differ as to the condition, the consequences of which are to be averted by the means recommended in common.

The extract is from the second fasciculus of Depaul's Clinical Lectures on Obstetrics, a most valuable volume, now in course of publication in Paris.

"In certain cases during pregnancy, but independent of labor, women find their garments wet with a profuse watery fluid coming from the vagina, and the physician is called to decide whether this is the amniotic liquid indicating rupture of the membranes. If it be amniotic, a peculiar faint odor will be noticed, and also the flow will occur from time to time, accompanying uterine contractions; besides, the finger lifting up the fetal head will permit a slight oozing, and if a small quantity of this liquid is collected in the hollow of the hand, its character is easily recognized; for if there are found suspended in it little whitish particles of fatty matter, it is amniotic, these particles being from the sebaceous coat which normally covers the body of the infant. So too, if the liquid is yellow or greenish, it is discolored by the meconium. But certain abundant watery discharges may occur, not coming from the body of the membranes, and are known as *false waters.* The generally received explanation of this formerly was that these discharges proceeded from a rupture of the chorion, a certain quantity of fluid being interposed between it and the amnion. But this amount is generally small, exhausting itself in a single discharge, and cannot produce those frequent and profuse flows which we are here considering, unless admitting with some authors that an endosmotic transudation through the amnion occurs, which will thus explain the abundance of the effused liquid.

"A much more rational explanation, which has been given by Naegle, and which is to-day demonstrated by undoubted facts, is that a certain part of the ovum is detached, and that the capillary vessels ruptured at this place of detachment permit the oozing of the serum of the blood which thus accumulates between the membranes and the uterine wall, and which may remain in its first position a greater or less length of time, until, either from a continual increase or from external violence, the detachment of the membranes continues, and the fluid arrives at the os and is suddenly discharged. It is easy to see that this fluid will be much greater as the extent of the detached surface increases; so too we readily perceive that there may be a continued oozing from this surface, and an external flow, until the ovum is applied anew to the uterine surface and the torn capillaries closed. In fact, if a patient suffering with this discharge is required to observe complete repose, the flow diminishes and then ceases. In some cases this phenomenon may be repeated several times in the same pregnancy; and I have observed women in whom this watery discharge has continued fifteen days, three weeks, a month, and even longer. With some this occurs in different pregnancies, and I have required patients to remain in bed several months when in preceding pregnancies the flow has resulted in premature labor, such termination not being rare in these circumstances.

"The liquid of the *false waters* is odorless, is clear, more transparent than the amniotic liquid, and presents all the characters of serum."

Still more interesting with reference to Dr. Chesnut's views is the report of a case (by M. Bailly, in Prof. Pajot's *Gazette Obstetricale,* March 20th, 1873) of *rupture of the membranes at eight months, and accouchement thirteen days after.* In his remarks upon it Bailly refers to a case, reported by Dr. Campbell, where pregnancy was prolonged seventeen days from the rupture of the membranes, labor ordinarily commencing from a few hours to three or four days after this accident occurs. He alludes to the explanation ordinarily given of these cases—viz: a serous collection between the chorion and the womb—as the source of the discharge; but states that the lessening of the size of the womb, the more distinct definition of parts of the fetus felt through the abdominal walls, and the continuousness of the discharge militate against this view. He finally remarks, however, that the only way to arrive at absolute certainty as to the source of the fluid is to use the test which we have already given in the extract from Depaul. T. P.

